# The Role of Diet and Lifestyle in Early-Onset Colorectal Cancer: A Systematic Review

**DOI:** 10.3390/cancers13235933

**Published:** 2021-11-25

**Authors:** Marta Puzzono, Alessandro Mannucci, Simone Grannò, Raffaella Alessia Zuppardo, Andrea Galli, Silvio Danese, Giulia Martina Cavestro

**Affiliations:** 1 Gastroenterology and Gastrointestinal Endoscopy Unit, Division of Experimental Oncology, IRCCS San Raffaele Scientific Institute, Vita-Salute San Raffaele University, 20132 Milan, Italy; puzzono.marta@hsr.it (M.P.); mannucci.alessandro@hsr.it (A.M.); zuppardo.raffaellaalessia@hsr.it (R.A.Z.); danese.silvio@hsr.it (S.D.); 2 Medical Biotechnologies Department, University of Siena, 53100 Siena, Italy; 3 Vita-Salute San Raffaele University, 20132 Milan, Italy; s.granno@studenti.unisr.it; 4 Gastroenterology Research Unit, Department of Experimental and Clinical Biochemical Sciences “Mario Serio”, University of Florence, 50121 Florence, Italy; a.galli@dfc.unifi.it

**Keywords:** young, colorectal neoplasia, obesity, smoking, risk factors, microbiota, epigenetics, LINE-1, antibiotics

## Abstract

**Simple Summary:**

This systematic review sifted through the exogenous dietary and lifestyle risk factors associated with early-onset colorectal cancer, going through the putative involvement of these exogenous risk factors in epigenetic and microbiota modifications. Given the burden of early-onset colorectal cancer and its globally increasing trend with scant literature on its pathogenesis, we believe it would be of benefit to highlight the importance of further systematic and large studies. Indeed, dietary and lifestyle modification could complement colorectal screening for early-onset colorectal cancer prevention.

**Abstract:**

The incidence of early-onset colorectal cancer, defined as colorectal cancer occurring in young adults under the age of 50, is increasing globally. Knowledge of the etiological factors in young adults is far from complete. Questionable eoCRCs’ exogenous factors are represented by processed meat, sugary drinks, alcohol, Western dietary pattern, overweight and obesity, physical inactivity, and smoking, though with heterogeneous results. Therefore, we performed a systematic review to summarize the current evidence on the role of diet and lifestyle as eoCRC risk factors. We systematically searched PubMed, Scopus, and EMBASE up to July 2021, for original studies evaluating diet, alcohol, physical activity, BMI, and smoking in eoCRC and included twenty-six studies. Indeed, the exogenous factors could represent modifiable key factors, whose recognition could establish areas of future interventions through public health strategies for eoCRC primary prevention. Additionally, we discussed the role of additional non-modifiable risk factors, and of epigenetic regulation and microbiota as mediators of the eoCRC triggered by diet and lifestyle.

## 1. Introduction

First described in the U.S.A. [1], an increase in the incidence of early-onset colorectal cancer (eoCRC) [2], defined as CRC before 50 years, was confirmed globally [3,4,5,6,7,8]. In Europe, eoCRC incidence significantly increased among individuals aged 20–39 years (y) in 12 out of 20 countries (Belgium, Germany, the Netherlands, the UK, Norway, Sweden, Finland, Ireland, France, Denmark, Czech Republic, and Poland) over the last 25 years. In the 40–49 y age group, the same increase was described in 8 out of 20 countries (the UK, Greenland, Sweden, Slovenia, Germany, Finland, Denmark, the Netherlands). On the other hand, a decreasing trend in Italy and no significant changes in the remaining European countries were described [6]. Indeed, data from 48 Italian cancer registries (2003–2014) covering 60% of the population and almost 15 millions aged 20–49 years confirmed the decreasing trend for both eoCRCs and late-onset CRCs (loCRCs) in Italy [7,8].

eoCRC has a distinct epidemiology, anatomical localization, histopathology, and clinical presentation compared to loCRC [9]. eoCRCs are typically located in the left colon (rectum, sigmoid colon, and descending colon) [10,11] and present more frequently at stage III–IV upon first diagnosis. They also tend to display a higher percentage of signet ring and mucinous histology as well as poor differentiation [2,10,12]. Most eoCRCs are sporadic in origin, with only a small percentage of eoCRC patients having a first-degree relative (FDR) with CRC [9,13]. Approximately 20% of eoCRCs carry a germline pathogenic variant of genes associated with CRC, half of which occur in DNA mismatch repair (MMR) genes associated with Lynch syndrome [14,15]. The remaining 80% usually have no family history of CRC [14].

Since hereditary gastrointestinal tumor syndromes only account for a minority of eoCRC cases, exogenous risk factors need to be investigated to better understand eoCRC pathogenesis. Alcohol intake, physical activity, red and processed meat, and a Western dietary pattern are well demonstrated loCRC risk factors [16,17,18,19,20,21,22,23,24,25]. Conversely, few studies, mostly case–control with only a handful of prospective studies, analyzed dietary, lifestyle, and anthropometric risk factors for eoCRCs and precursors [26,27,28,29,30,31,32,33].

This systematic review summarizes the current evidence on the role of diet and lifestyle as risk factors in eoCRC. The exogenous factors could represent modifiable key factors, whose recognition could establish areas of future interventions through public health strategies for eoCRC primary prevention. Additionally, we contextualize our findings, by presenting the role of additional non-modifiable risk factors. We also discuss the role of epigenetic regulation and microbiota as mediators of the eoCRC triggered by diet and lifestyle.

## 2. Materials and Methods

This systematic review was conducted according to the Preferred Reporting Items for Systematic Reviews and Meta-Analyses (PRISMA) guidelines. The authors M.P. and A.M. conducted the systematic literature search on Pubmed, Scopus and Embase until July 2021 using the terms “diet”, “meat”, “vegetables”, “milk”, “cheese”, “smoking”, “cigarette”, “e-cigarette”, “dietary pattern”, “BMI”, “obesity”, “overweight”, “dairy”, “dairy products”, “drinks”, “alcohol”, “wine”, “physical activity”, “exercise”, “physical exercise”, “sedentary”, “sedentary lifestyle”, “risk factor”, “early”, “young”, “onset colorectal”, “cancer”, and “neoplasia”, combined with Boolean terms.

**Figure 1:**
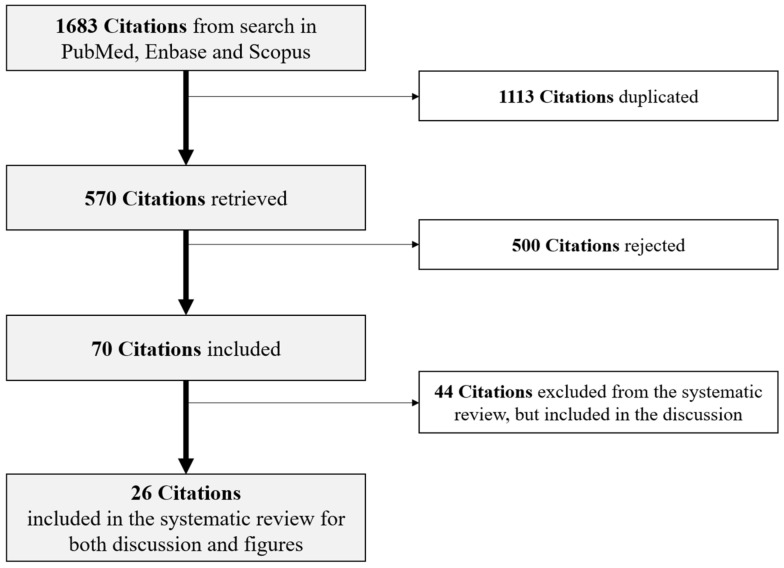
Flow chart showing screening and inclusion of the eligible studies.

After deduplication, M.P., A.M. and G.M.C. initially selected the articles through title and abstract screening, followed by full-text reading for each title/abstract deemed to be relevant. Studies were eligible to be included in this systematic review if they were performed on CRC or advanced colorectal neoplasia (ACRN) diagnosed under the age of 50 years, published as original articles, reported relative risks (RR) or hazard ratios (HRs), or odds ratios (ORs) for the association of the exogenous risk factors listed above with eoCRC. Conversely, we excluded studies not reporting specific results for populations with CRC below 50 years, non-English articles, reviews, and case report articles, abstract only. Twenty-six studies were finally included in this systematic review for relevant data extraction (Figure 1): year of publication, study design, country where the research was performed, size sample with the number of eoCRC cases (and/or ACRN) and controls (if present), the age range or mean age ± standard deviation (SD), sex, list of the exogenous risk factors analyzed for each article, and ORs/HRs/RRs (±95% CI) for eoCRC. We then collected this information on a Microsoft Excel spreadsheet.

## 3. Diet and eoCRC

A robust body of literature demonstrated that a diet high in red and processed meat with low fibers consumption represents a key risk factor for loCRC [23,34,35,36,37,38,39,40]. Moreover, a protective effect of a high intake of vegetables and fruit was demonstrated in loCRC. [16,36,38,39,40,41,42,43,44]. The results were instead too disparate to support any protective effect of fish and omega-3 on loCRC [36,40,45,46,47,48,49].

Indeed, a diet rich in red and processed meat leads to colorectal carcinogenesis through gut epithelial damages and proliferation, DNA damage, and genotoxicity by means of heterocyclic amines, polycyclic aromatic hydrocarbons (PAH), and N-nitroso compounds (NOCs) from nitrate/nitrite added to processed meat as preservatives [50,51,52]. Moreover, red meat heme iron catalyzes NOC formation and lipid peroxidation [53,54]. Dietary fibers seem to exert their protective effect on loCRC development by acting on the intestinal microbiota, enriching Lactobacillus spp. and butyrate-producing bacteria, and increasing short-chain fatty acid production [55]. The latter act in turn through the modulation of regulatory T cells and the regulation of gene expression through epigenetic mechanisms [55,56].

These exogenous risk factors vary along with different sub-sites of the colorectum. A significant positive association with loCRC risk increasing from cecum to transverse colon was found for alcohol intake and from cecum to rectum for processed red meat [24]. Conversely, whole grains and cereal fibers were inversely associated with loCRC risk, with that association increasing from cecum to rectum. Similarly, loCRC patterns along colorectal subsites were reported in a sub-group of 901 “younger-onset” CRC, with less than 60 y, too.

In contrast with the consistent literature available on dietary factors and loCRC, few studies evaluated the role of diet and alcohol habits in eoCRC pathogenesis during the last 13 years (Table S1).

### 3.1. Foods and eoCRC

**Figure 2:**
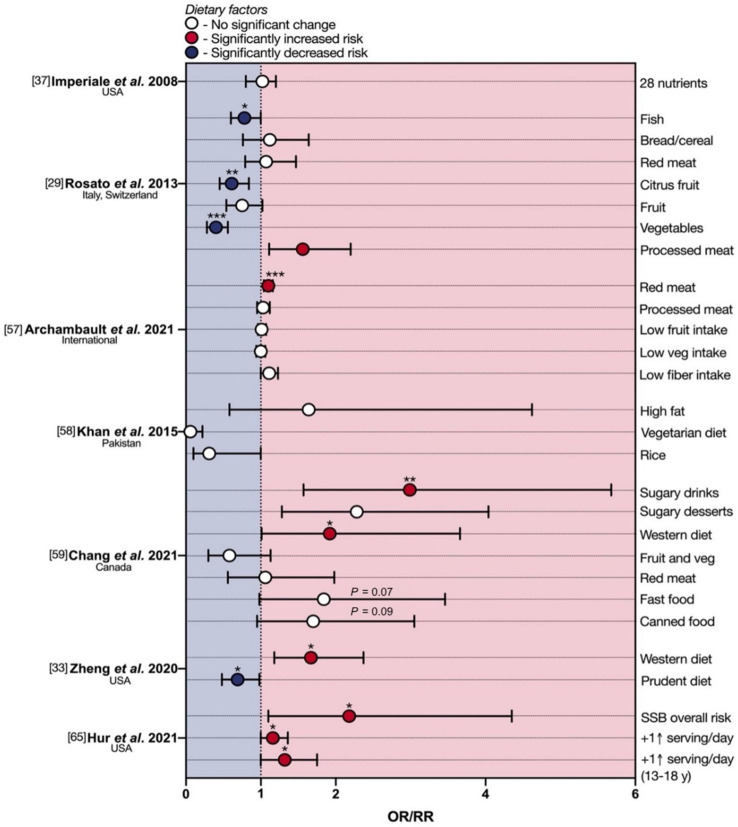
Protective and deleterious effects of diet in early-onset colorectal cancer. eoCRC—early-onset colorectal cancer; Met–meta-analysis; OR—odds ratio; RR—relative risk; SSB—sugar-sweetened beverage. * *p* < 0.05; ** *p* < 0.01; *** *p* < 0.001.

The first multi-center retrospective case–control study was performed by Imperiale et al. [37] on a small sample of North American individuals with advanced colorectal neoplasia (ACRN), defined as the presence of colorectal adenocarcinoma, polyps with high-grade dysplasia or villous histology, or tubular adenomas ≥1 cm. Twenty ACRN (of which 11 eoCRCs) and 54 age-matched controls were surveyed by means of a validated food-frequency questionnaire (1998 block food-frequency questionnaire [34]), including questions on ethanol consumption. No differences were observed between ACRN cases and controls in any of the 28 nutrients of the block food frequency questionnaire, including total calories, daily fat consumption in grams per day, folate consumption in micrograms per day, percent of calories from fat, protein, and carbohydrates (Figure 2).

A second multi-center case–control study was conducted on an Italian/Swiss population of 329 eoCRCs ≤ 45 y, of which 208 colon cancers and 121 rectal cancers, compared to 1361 age-matched controls [29]. A validated food-frequency questionnaire on the usual consumption of 78 foods assessed dietary and alcohol habits referred to the two years prior to eoCRC diagnosis [35]. Multiple logistic regression analysis showed a significant increase in eoCRC risk with high intake of processed meat (odds ratio (OR) 1.56 for high tertile of intake vs. the lowest one; Figure 2); a significant inverse association was found between eoCRC and high consumption of vegetables (OR 0.4), citrus fruit (OR 0.61) and fish (OR 0.78) (Figure 2). A high tertile intake of red meat (OR 1.07), bread and cereals (OR 1.12), fruit (OR 0.75), or olive oil (OR 0.78) did not reach statistical significance.

Archambault and colleagues recently pooled and analyzed data from 13 population-based studies, including 3767 eoCRCs and 4049 age- and sex-matched controls, via multivariable and multinomial logistic regression for association with several risk factors [57]. The results showed that eoCRC may be significantly associated with greater red meat intake (OR 1.10). In contrast, no differences in OR were observed for fruit, vegetable, processed meat, and total dietary fiber intake.

Few dietary practices and alcohol habits were also analyzed as non-quantitative data in a Pakistani single-center case–control study of 74 eoCRCs and 148 age- and gender-matched controls [58]. Significant protective effects of rice and rice powder, being vegetarian, as well as a non-high fat diet, were found (Figure 2).

Chang et al. performed a retrospective case–control study on a small population of 175 eoCRCs, analyzing a large list of exogenous factors in a systematic manner [59]. Greater consumption of sugary drinks (≥7/w; OR 2.99) and sugary desserts (3–6/w; OR 2.28), as well as a higher Western-like dietary pattern score (quartile 4, OR 1.92), were associated with an increased eoCRC risk. Statistically significant associations were not observed for fruits, vegetables, high-fiber foods, red meat, or processed meat, although greater vegetable consumption showed a tendency toward lower risk (*p* = 0.08). In addition, more frequent consumption of fast food (≥2/w; OR 1.84) and canned food (≥3/w; OR 1.70) showed suggestive associations with increased eoCRC risk.

Zheng et al. [33] performed the first multicenter prospective cohort study on dietary patterns and risk of early-onset high-risk adenomas (eoHRA) as eoCRC precursors in a large sample of 29,474 women followed between 1991 and 2011 as part of the Nurses’ Health Study II (NHS II) by means of a quadrennial food frequency questionnaire. A total of 375 eoHRAs were found during colonoscopy surveillance, with the highest quintile of Western diet consumption as eoHRA risk factor (OR 1.67) and the highest quintile of prudent diet as a protective factor (OR 0.69) (Figure 2). Moreover, the Western diet was associated with advanced adenomas of the distal colon and rectum (OR 1.65), consistent with the typical eoCRC localization in the left colon and rectum [10,11]. These two dietary patterns have been described as exogenous factors associated with the prevention or predisposition of loCRC, too: respectively, the ‘healthy’ pattern (high in fruits, vegetables, and whole grains or legumes, fish, and low-fat milk or dairy products) and the ‘unhealthy’ or ‘Western dietary’ pattern (high in red and processed meat, sugary drinks, refined grains, desserts) [60]. Above all, the Western diet is one of the most important loCRC risk factors [51,58,61], and it is associated with the development of high-risk rectal adenomas later in life if started during adolescence [62]. Conversely, the Mediterranean diet demonstrated a protective role in CRC development [19,63], confirmed in the Italian section of the EPIC cohort [64].

A recent prospective study by Hur and colleagues [65] also interrogated the NHS II female cohort to assess the relative risk of eoCRC linked with the intake of sugar-sweetened beverages (SSBs) during both adulthood and adolescence (13–18 years). They demonstrated a doubled risk of eoCRC in women who consumed ≥2 servings of SSBs per day during adulthood, with a 16% higher risk (RR 1.16; 95% CI 1.00 to 1.36) for each serving/day increase. Moreover, each serving/day increment of SSBs intake during adolescence was associated with a 32% higher risk of eoCRC. The authors also reported that the replacement of each serving/day of SSB consumed during adulthood with that of artificially sweetened beverages, coffee, low-fat milk, or total milk was, conversely, associated with a 17–36% lower risk of eoCRC (Figure 2).

No study has so far analyzed the consumption of milk and dairy products as a risk factor for eoCRC, although their protective effect on loCRC in three meta-analyses of observational studies was demonstrated [40,66,67]. Indeed, calcium is considered the main protective nutrient in dairy products. It may act through binding to secondary bile acids and ionized fatty acids, reducing their carcinogenic effects on the colorectal epithelium [68]. Moreover, calcium may promote both differentiation in normal cells and apoptosis of transformed cells through cell signaling modulation [69].

### 3.2. Drinks and eoCRC

**Figure 3:**
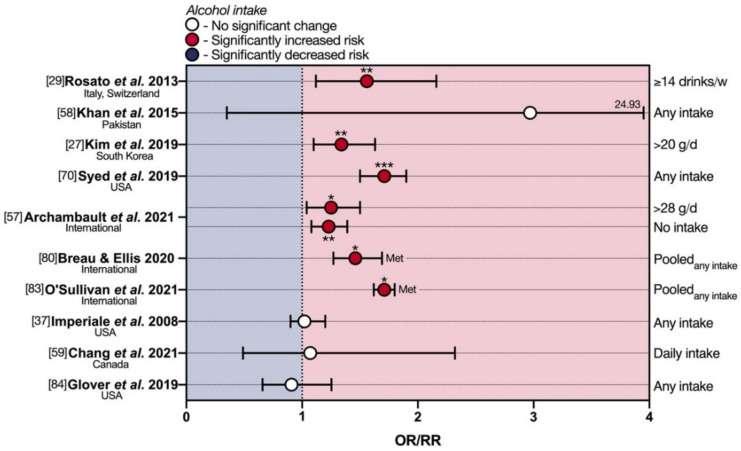
Deleterious effects of alcohol in early-onset colorectal cancer. eoCRC—early-onset colorectal cancer; Met—meta-analysis; OR—odds ratio; RR—relative risk. * *p* < 0.05; ** *p* < 0.01; *** *p* < 0.001.

Concerning alcohol habits (Figure 3), Rosato et al. [29], showed a significant increase in eoCRC risk with an alcohol consumption ≥14 drinks/week (OR 1.56) at multiple logistic regression, while a detrimental effect of alcohol intake was observed only at univariate analysis in the Pakistani eoCRC population [58]. A multi-center retrospective cross-sectional study by Kim et al. [27] confirmed alcohol intake ≥20 g/day as an exogenous risk factor for ACRN (of which 14 eoCRCs) only in the 30–39 y group (OR 1.34). This is in line with recent findings by Syed and colleagues (OR 2.46) [70], who performed a population-based cohort study on 5710 eoCRCs aged 25–49 from the Explorys Database (Figure 3). These results reported in eoCRCs are consistent with those of nine meta-analyses of observational studies [71,72,73,74,75,76,77,78,79], showing a significantly increased loCRC risk, with a dose-dependent effect of alcohol.

Similarly, the pooled analysis by Archambault et al. [57] suggests that eoCRC may be significantly associated with heavier alcohol use (>28 g/day of alcohol; OR 1.25). It is interesting to note that the authors also found a significant increase in the risk of eoCRC with alcohol abstinence (OR 1.23).

Breau and Ellis conducted a systematic review and meta-analysis of epidemiologic studies to identify lifestyle and clinical risk factors associated with young-onset colorectal adenomas and cancer (yCRAC) in adults below 50 years [80]. Three of the studies reviewed therein examined the association between advanced yCRAC diagnosis and alcohol intake [27,81,82]. A pooled OR of 1.46 was reported, suggesting significantly increased risk, although the three studies employed different criteria to define excessive alcohol consumption. Another recent meta-analysis of 14 studies by O’Sullivan et al. also linked greater alcohol consumption (vs. abstinence) to enhanced eoCRC risk (relative risk (RR) 1.71) [83].

In contrast to the above, several lower-powered reports have failed to identify any associations between alcohol use and eoCRC risk. Imperiale et al. found no differences in alcohol consumption between ACRN cases and controls [37], and neither did Chang et al. [59] (Figure 3). A retrospective analysis of a large population-based database by Glover and colleagues also found no association of eoCRC risk with alcohol abuse (not further specified) [84]. Data were acquired from 26 healthcare systems spread over 50 U.S. states, comparing individuals with CRC below 40 years with age-matched controls without CRC via multivariate analysis.

A putative mechanism for chronic alcohol intake-promoted colorectal carcinogenesis is the induction of intestinal dysbiosis. Indeed, ethanol lowers the abundance of Bacteroidetes and Firmicutes, enriches Proteobacteria and Actinobacteria, leading to intestinal hyperpermeability, increased translocation of gram-negative endotoxins, and systemic inflammation [85,86,87,88]. Intestinal dysbiosis may also enhance ethanol oxidation, with intra-colorectal levels of acetaldehyde exceeding the minimum concentrations necessary for carcinogenesis [89].

## 4. Physical Activity and eoCRC

The notion that a sedentary lifestyle confers an enhanced risk of loCRC is well established [90,91]. A mechanism through which physical inactivity could exert its harmful effects is represented by gut dysbiosis [92,93]. Indeed, regular exercise increased the Bacteroidetes-to-Firmicutes ratio in rat models [94,95], and moderate physical activity revealed a higher abundance of health-promoting bacterial species, including *Faecalibacterium prausnitzii*, *Roseburia hominis*, and *Akkermansia muciniphila*, in active women [92].

**Figure 4:**
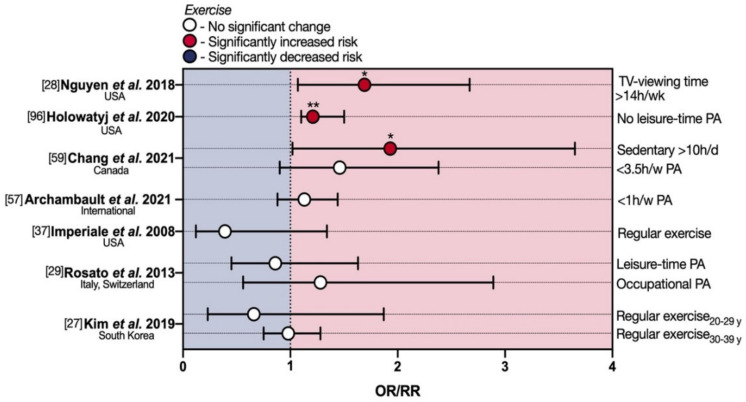
Protective and deleterious effects of physical activity in early-onset colorectal cancer. eoCRC—early-onset colorectal cancer; OR—odds ratio; PA—physical activity; RR—relative risk. * *p* < 0.05; ** *p* < 0.01.

A prospective cohort study by Nguyen et al. [28] investigated, in the NHS II eoCRCs population, weekly sedentary TV viewing time, a surrogate of physical inactivity, as a risk factor for eoCRC. Multivariate analysis, after adjusting for other CRC risk factors primarily BMI and for weekly physical energy expenditure, found a significant 1.69-fold increase in eoCRC RR associated with weekly TV viewing times of 14 h or more and a slightly significant increased RR of 1.12 for weekly times ranging from 7.1 to 14 h. This association was more pronounced for rectal cancer (RR 2.44 for weekly TV viewing times of 14 h or more) (Table S1; Figure 4). Moreover, overweight/obese subjects (BMI ≥ 25), those performing less physical activity (<15 metabolic equivalents of task-hours/week), and ever-smokers showed an increased eoCRC risk, with the strongest interaction between high BMI and sedentary TV viewing time. Other types of sitting time at home, including mealtime or time spent at a desk, and sitting away from home were evaluated without finding a clear increased eoCRC risk.

This is in line with a recent population-based cohort study [96], which employed geospatial autocorrelation in order to identify ‘hotspots’ of eoCRC mortality across U.S. territory on a female population. Spearman rank correlation showed that physical inactivity, defined as reporting no leisure-time physical activity at or after age 20, was positively correlated with hot spot areas of eoCRC mortality among U.S. women (ρ 0.21). In further support of these lines of evidence, Chang et al. also showed that increased sedentary time (≥10 vs. <5 h/day) was associated with a statistically significant enhanced risk of eoCRC (OR 1.93) [59]. Being less physically active trended toward higher risk in the same study, despite a lack of statistical significance.

Despite these reports linking physical activity (or lack thereof) to eoCRC, an approximately equal weight of evidence to the contrary exists at present. Archambault and colleagues [57] found no difference in eoCRC with sedentary lifestyle defined as <1 h/w of moderate/vigorous physical activity, leisure time, and undifferentiated activities. The 2008 multi-center retrospective case–control study by Imperiale and colleagues [37] failed to identify an association between eoCRC and physical activity, defined as regular exercise, even if not further specified. Although the measured OR showed a trend toward reduced risk for active individuals (0.39), this did not reach statistical significance (Table S1; Figure 4), probably due to the small sample including 20 overall cases of ACRN, 11 of which were categorized as true eoCRCs. Similarly, the Italian/Swiss case–control study by Rosato et al. did not identify a statistically significant reduction in eoCRC risk linked to both occupational physical activity (evaluated as mainly sitting, mainly standing, intermediate, heavy, strenuous) and leisure-time physical activity (hours per week) both at 30–39 years (OR for highest vs. lowest level of physical activity 1.28 and 0.86, respectively) [29], (Table S1; Figure 4). More recently, a multi-center retrospective cross-sectional analysis of a mixed-gender South Korean eoCRC population produced similar results [27]. Indeed, the multivariate OR for ACRN was 0.66 for regular exercise (defined as moderate or vigorous for ≥3 times/week) in the 20–29 y group and 0.98 in the 30–39 y group, without reaching statistical significance.

## 5. Obesity and eoCRC

**Figure 5:**
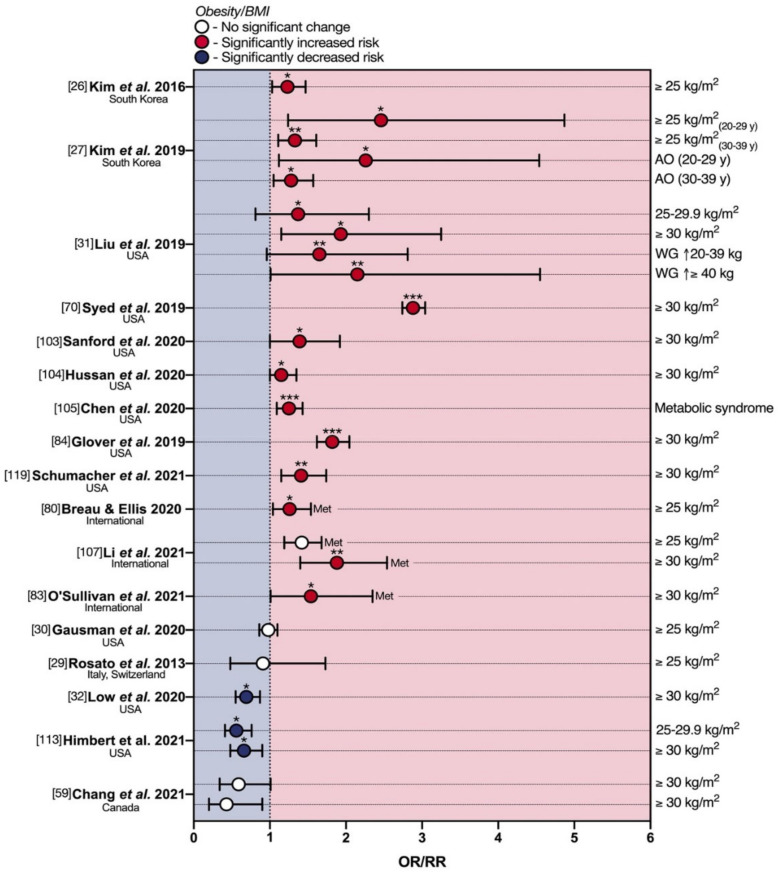
Protective and deleterious effects of obesity in early-onset colorectal cancer. AO—abdominal obesity; BMI—Body mass index; eoCRC—early-onset colorectal cancer; Met—meta-analysis; OR—odds ratio; RR—relative risk; SSB—sugar-sweetened beverage; WG—weight gain. * *p* < 0.05; ** *p* < 0.01; *** *p* < 0.001.

Excessive body weight is one of the most universal risk factors for several types of disease and, as for physical activity and dietary factors, there is a well-established association with loCRC [90]. In addition to the known association with the metabolic syndrome and, therefore, with hyperinsulinism [97,98], obesity is involved in colorectal carcinogenesis through modifications of the gut microbiota [99], leading to increased levels of microbial-derived pro-inflammatory molecules (lipopolysaccharide, increased acetate, and reduced butyrate) that in turn impair the intestinal barrier [100]. Other dysbiotic mechanisms of action are represented by epigenetic remodeling [101,102], and alterations in the gut microbial metabolites (deoxycholic acid, a secondary bile acid produced by Gram-positive) causing DNA damage. Over recent years, several studies have also investigated this risk factor in the context of eoCRC (Table S1; Figure 5).

In 2016, drawing from a male/female South Korean cohort of 59,782 individuals, Kim et al. retrospectively assessed body mass index (BMI) in 564 cases of ACRN (25 eoCRCs) [26]. Patients with BMI ≥ 25 kg/m^2^ displayed a statistically significant 1.23-fold increase in multivariate OR for ACRN. This trend is mirrored in the subsequent cross-sectional analysis by Kim et al., where the association was quite strong, being observed as a significant increase in eoCRC risk for BMI ≥ 25 kg/m^2^ in both 20–29 y (OR 2.46) and 30–39 y group (OR 1.33) (Table S1, Figure 5) [27]. The authors further assessed pure abdominal obesity as an eoCRC risk factor, with a notable peak in the 20–29 age group for abdominal obesity (OR 2.26).

This phenomenon has also been observed in the U.S. population, on which the vast majority of available studies focus. Liu et al. probed for an association between eoCRC RRs and BMI in a prospective study on NHS cohort II [31]. For both ‘overweight’ (BMI 25–29.9 kg/m^2^) and ‘obese’ (BMI ≥ 30 kg/m^2^) thresholds, the multivariate RRs were significantly enhanced 1.37- and 1.93-fold, respectively (Table S1, Figure 5). The authors also investigated the effects of weight gain after 18 years of age, showing that weight gains of either 20–39.9 Kg or ≥40 Kg were associated with statistically significant increases in RR of 1.65 and 2.15-fold, respectively. This is in line with observations made by Syed et al. in a 2019 male/female population-based cohort analysis, on 5710 eoCRC cases [70], from the Explorys database. In this study, the association with BMI ≥ 30 kg/m^2^ was quite strong. Indeed, the authors reported a significant 2.88-fold increase in multivariate OR for first-incidence eoCRC development in obese individuals. In further support, the association appears to hold even in self-reported eoCRC cases, as shown by Sanford et al. in a 2020 U.S.A.- and questionnaire-based follow-up study [103]. For obese patients (BMI ≥ 30 kg/m^2^), the multivariate OR for eoCRC was significantly increased 1.39-fold. A 2020 cohort study by Hussan et al. [104], assessed rates of obesity at eoCRC resection over time in a U.S.-based case–control study, identifying a significant 15.3% annual increase in obesity at the time of colon resection throughout the 2002–2013 period. Conversely, Chen and colleagues investigated metabolic syndrome co-morbidity in eoCRC [105]. This nested case–control study comprised 4673 male/female instances of eoCRC, with ages ranging from 18 to 50 and a median age of 43. The presence of metabolic syndrome was associated with a significant 1.25-fold increase in multivariate OR for eoCRC. Glover et al. [84] also found that eoCRC (<40 y) was significantly associated with obesity (OR 1.82) at multivariate analysis. Recently, Schumacher et al. performed a retrospective population-based case–control study at Kaiser Permanente Southern California, an integrated health care delivery system serving over 4.4 million members. Obesity (OR 1.41) was significantly associated with an increased risk of eoCRC at multivariate analysis, especially for early-onset colon cancer (OR 1.56). No significant association was reported between obesity and early-onset rectal cancer. Four of the studies evaluated in combination by Breau and Ellis [80] identified obesity (BMI ≥ 25 kg/m^2^) as a risk factor for advanced yCRAC, with a pooled OR of 1.26 [27,81,82,106]. Through a similar approach, Li et al. [107] performed a meta-analysis of six studies analyzing elevated BMI as a risk factor for eoCRCs (age ≤ 55 years) [70,108,109,110,111,112]. They found that overweight and obesity (BMI ≥ 25 kg/m^2^) were associated with a 42% increased risk of CRC compared with normal weight (OR 1.42). When setting the age cutoff at 50, the OR was 1.38. A substantially stronger excess risk was observed for obesity (OR 1.88) than for overweight (OR 1.32). Sensitivity analyses with a cutoff age of 50 years showed very similar results. The 14-study 2021 meta-analysis by O’Sullivan and colleagues also confirmed obesity as a risk factor (RR 1.54) [83].

Whilst there appears to be a clear trend toward an association between excess body weight and eoCRC, some reports contradicted these results. In 2018, a U.S.A.-based, single-center retrospective case–control [30] investigated 269 cases of eoCRC in a mixed-gender population aged 20–49. Amongst several risk factors, the authors also assessed overweight by BMI, finding no statistically significant differences in multivariate OR for eoCRC (0.98-fold) if compared with age-matched healthy controls (Figure 5). The previously mentioned Italian/Swiss population study by Rosato et al. [29] reported a slightly reduced eoCRC risk with BMI ≥ 25, but with no statistical significance (OR 0.91) (Figure 5). Moreover, three recent studies went as far as showing a diametrically opposite finding: a protective effect of high BMI on eoCRC development [32,59,113]. The first was a multi-center retrospective case–control study by Low et al. based on a U.S. mixed-gender population of 651 cases aged 18–49 [32]. For both overweight and obese BMIs, the authors identified a statistically significant 0.69-fold decrease in eoCRC multivariate OR. Interestingly, the data also showed that an underweight BMI conferred a significant 1.87-fold increase in eoCRC risk (Figure 5). The second was the U.S.A.- and Germany-based prospective cohort study by Himbert and colleagues, comparing eoCRCs with loCRCs [113]. Patients with eoCRC were less likely to be overweight and obese compared to loCRCs at multivariate analysis (OR 0.56 and 0.66 respectively), while underweight eoCRC patients had an OR of 1.08. Finally, the previously mentioned report by Chang et al. [59] showed a suggestive inverse correlation (*p* = 0.06) between obese BMI and eoCRC risk, both in early adulthood (20 s) and 2 years before diagnosis, with respective ORs of 0.43 and 0.59.

## 6. Smoking and eoCRC

**Figure 6:**
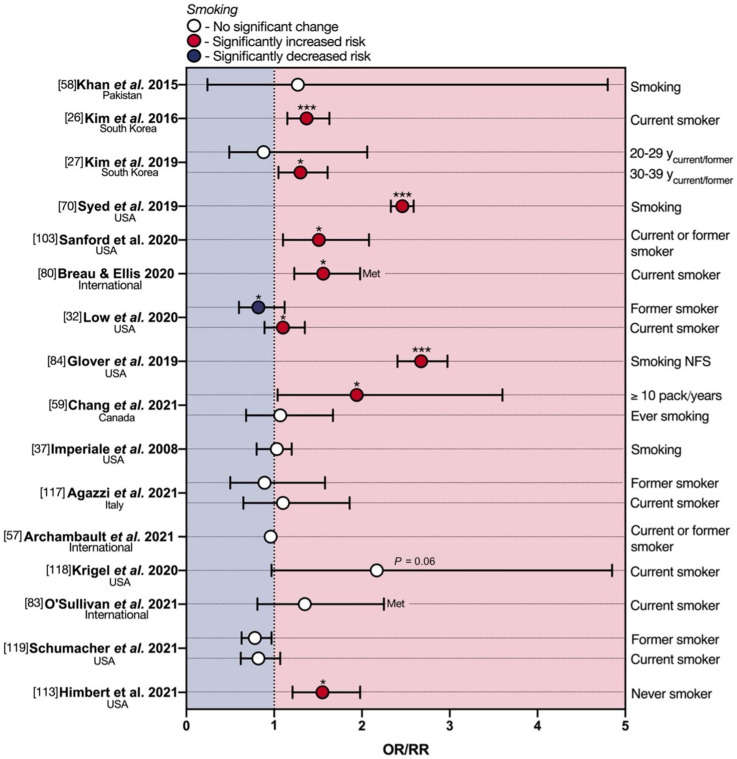
Protective and deleterious effects of smoking in early-onset colorectal cancer. eoCRC—early-onset colorectal cancer; Met—meta-analysis; NFS—not further specified; OR—odds ratio; RR—relative risk. * *p* < 0.05; *** *p* < 0.001.

Tobacco smoking is another critical risk factor in the development of many cancers and has been previously associated with loCRC [114,115,116]. Mounting evidence also suggests a consistent link to eoCRC (Table S1, Figure 6).

The above-discussed 2015 monocentric case–control study by Khan et al. [58] investigated smoking status (only defined as yes or no) in a cohort of 74 Pakistani male and female patients diagnosed with eoCRC in age range of 14–85 (median age ~42). Only the univariate OR for smoking reached statistical significance, showing an increased eoCRC risk of 2.12-fold. However, when the analysis was adjusted for multivariable risk, significance was lost (1.27-fold). In support of the univariate analysis by Khan et al., both South Korean studies by Kim et al. mentioned above showed similar results, referring to ACRN risk, however. Indeed, the 2016 study by Kim et al. found a statistically significant 1.37-fold enhancement in the multivariate OR of ACRN for current smokers [26]. The 2019 study by Kim et al. [27], which segregated patients into age groups, showed, on the other hand, a significantly increased multivariate risk of ACRN for current or former smokers only in the 30–39 y group (OR 1.30), but not in those aged 20–29 y (OR 0.88). The population-based cohort analysis by Syed and colleagues [70] also demonstrated a statistically significant 2.46-fold increase in eoCRC risk for smoking status (only defined as yes or no), as did the follow-up study on self-reported eoCRC diagnosis by Sanford et al. [103], with a significant 1.51-fold change in OR for current or former smokers. Following the above, four studies [27,81,82,106], analyzed in combination by Breau and Ellis [80], also found an association between current, regular smoking, and an advanced yCRAC diagnosis, with a pooled OR of 1.56.

These lines of evidence are further mirrored in the 2020 case–control study by Low et al. [32], albeit less markedly. The data indicated a modest but statistically significant 1.10-fold increase in eoCRC risk for current smokers. Interestingly, the authors also found a slightly protective effect of no longer smoking, with former smokers enjoying a significant 0.82-fold decrease in OR. Glover and colleagues [84] instead showed a more substantial eoCRC (<40 y) risk enhancement with smoking (not further specified) at multivariate analysis (OR 2.675). Whilst Chang et al. found no differences in eoCRC risk between ever- vs. never-smoking patients, those in the first tertile of pack-years had a significantly elevated risk compared to never smokers (OR 1.94) [59].

Perhaps counterintuitively, there is also a meaningful body of evidence dismissing smoking as a risk factor for eoCRC (Figure 5). Imperiale et al. did not find any significant effect of smoking (not further specified) on eoCRC risk [37]. Agazzi and colleagues demonstrated that the smoking status was not significantly associated with either the presence of adenomas or eoCRC in a 2021 study, even accounting for bias related to pooling of eoCRC and adenomas cases [117]. In line with this, no statistically significant differences were observed between eoCRCs and healthy age and sex-matched controls in pack/year smoking among current and former smokers in the above-discussed report by Archambault et al. [57]. In a 2020 single-center retrospective observational study on a small sample of 48 advanced colorectal neoplasia, Krigel et al. also failed to demonstrate smoking status as a risk factor for eoCRC [118]. Whilst smoking trended as a risk factor (RR 1.35) according to O’Sullivan and colleagues, the correlation did not reach significance [83]. Likewise, no significant association between eoCRC and current or former smoking was found by Schumacher et al. [119]. The prospective multicenter study by Himbert et al. discussed above even showed eoCRC patients as significantly more likely to be never smokers compared to loCRC patients (OR 1.55), although this is a solitary finding [113].

## 7. The Relationship of Other Risk Factors for eoCRC with Diet and Lifestyle

The risk factors discussed so far are modifiable, but others are not. Well-established non-modifiable risk factors include age, family history, race, and hereditary gastrointestinal syndromes [120,121,122,123,124,125,126,127]. The combination of a congenital (un-modifiable) predisposition with the exposure to environmental (modifiable) risk factors, especially at a young age [128], can increase the risk of CRC to a greater extent than each factor alone through various mechanisms, including epigenetic changes and intestinal dysbiosis.

### 7.1. Epigenetics and eoCRC

Before the age of 50 years, other mechanisms besides epithelial senescence are likely responsible for dysplasia and cancer. Specifically, aberrant epigenetic processes may be one of the mechanisms through which diet and lifestyle habits promote not only loCRC but also eoCRC. Epigenetic alterations include DNA hypomethylation, CpG island promoter hypermethylation, small non-coding RNAs, and histone modification. Among these, global DNA hypomethylation is an early epigenetic alteration in loCRC. It has been associated with proto-oncogenes activation [129], chromosomal instability [129,130,131], and can be indirectly measured by assessing the methylation status of tumor long interspersed nucleotide element-1 (LINE-1) repeat sequences.

In loCRCs, alcohol abuse, low folate, and vitamin A intake were associated with higher promoter hypermethylation of genes involved in CRC development (i.e., APC, MLH1, p14, p16) [132,133]. Moreover, LINE-1 hypomethylation was more common among CRC patients referring to a lower folate intake and higher alcohol [134]. Other exogenous risk factors associated with LINE-1 hypomethylation are represented by high BMI, low physical activity [135], and smoking [136].

Although it has been suggested that LINE-1 hypomethylation is associated with eoCRC, this association has been poorly analyzed yet. The first study by Antelo et al. [137] reported lower LINE-1 methylation levels in eoCRCs than loCRCs and Lynch syndrome-CRC, suggesting a potentially unique and distinct feature of eoCRCs. A recent study [138] confirmed these results, additionally showing higher colorectal cancer-associated mortality and a progressive decreasing trend with younger age of CRC. Indeed, patients with CRC aged 50–54, defined as having “intermediate-onset colorectal cancer”, showed LINE-1 hypomethylation less commonly than eoCRC but more frequently than older patients with CRC. Nevertheless, this association between eoCRC and tumor LINE-1 hypomethylation recently described in the literature suggests that exposure to these exogenous risk factors could have an important role in eoCRC development, too.

One of the mechanisms by which dietary fibers protect from CRC is the production of short-chain fatty acids. One such fatty acid, butyrate, is the byproduct of microbiota fermenting species. In vitro, butyrate modulates the cell cycling of both crypt stem cells and malignant colonocytes [139]. Butyrate could slow the proliferation of CRC cells and induce their apoptosis [140]. The most likely mechanism for these effects is higher acetylation of histones [141]. Some studies have also claimed a connection between butyrate and the stability and translation of micro-RNA [142], specifically those micro-RNAs that modulate the expression of oncogenes, such as PTEN, TP53, and CDKN1A [143,144]. The biological link between histone acetylation, butyrate, and eoCRC is only speculative at this stage. Further research is needed to evaluate the impact of the diet on histone modifications.

Small non-coding RNAs include endogenously expressed transcripts that regulate several cellular processes, including cancer development and progression [145]. Based on their size and function, they are classified into several subtypes, including micro-RNA (mi-RNA), small interfering RNA (si-RNA), piwi-interacting RNA (pi-RNA), and others. In general, mi-RNAs regulate gene expression by binding the 3′-untranslated region of their target messenger RNA, thus inhibiting translation of the messenger RNA. The aberrant expression of mi-RNAs can contribute to CRC onset [146], progression, and metastasis [147]. The role of mi-RNAs appears context-specific: each mi-RNA has a unique potential to promote (or suppress) tumor development [148]. For example, KRAS-mutated CRC overexpress miRNA-425-5p, which targets genes in the EGFR pathway, thus accounting for resistance to anti-EGFR therapies, at least in part [149]. A recent study concluded that miR-139-5p restrains CRC progression and invasion via the *Ras* and *Wnt* signaling pathways [150]. Our understanding of mi-RNAs in eoCRC is limited by the lack of studies focused on mi-RNA and eoCRC. There is sufficient evidence on mi-RNA in CRC to hypothesize that mi-RNA could contribute to eoCRC as well. However, this compelling hypothesis requires further studies to identify the mi-RNAs that are specifically responsible for the younger age of onset.

There is increasing evidence that epigenetic information can reach across generations independent of genetic transmission [151]. Recent studies have described some mechanisms by which non-genetic information is relayed independently of the nucleotide sequence. Epigenetic inheritance can occur via shared somatic modifications (shared diet and environment in the same household), via gamete-based processes, but also via the transmission of DNA methylation, histone modifications, and expression of non-coding RNAs [152]. At the molecular level, several environmental agents can directly influence phenotypic variation, genetic variation, and inheritance [153], mostly via the transmission of DNA methylation and histone modifications across generations [154]. Family history is a strong risk factor for the development of CRC, especially at a young age [83]. However, inherited CRC syndromes do not fully explain the familial component of eoCRC [15,155,156,157,158]. Non-Mendelian epigenetic inheritance could account for those cases with unclear inheritance patterns. Nevertheless, it is worth mentioning that the evidence on the epigenetic inheritance of CRC is still poor, and the idea of a non-Mendelian inheritance of eoCRC, although captivating, is still speculative. Additional studies need to identify the epigenetic basis of eoCRC, which remains largely elusive at this time.

### 7.2. Microbiota, Early Exposure to Risk Factors, and eoCRC

It has been speculated that exposure to risk factors should occur in the very early life to account for the epidemiological rise in eoCRC incidence, particularly in the early life of white children [159]. Three observations support this hypothesis. First, the epidemiological increase in eoCRC demonstrates a strong birth-cohort effect [9,160]. Second, the adenoma-carcinoma sequence has a long latency period between exposure and CRC [161,162,163,164]. Third, the epidemiological increase in eoCRC is largely fueled by an increase in rectal cancer in white people, with the steepest rise after 2012, while blacks and pacific islanders did not experience any increase [165,166]. These considerations have led investigators to explore pediatric exposure to antibiotics as a risk factor for eoCRC [163,167]. Previous studies reported an increased risk of loCRC after repeated exposure to penicillin and other [168,169], likely due to alterations in the microbial composition and the nitrogen [170]. Pediatric studies have noticed that not only do white children receive more antibiotics than black children [171], but they also receive more inappropriate prescriptions for viral infections [172]. However, such studies only took place after 2000. Similar results should be confirmed in adults born after 1950 to explain the aforementioned birth-cohort effect. Such evidence demands a deeper understanding to draw meaningful conclusions.

The question of whether the microbiota could affect the development of CRC in younger adults remains complex. On one hand, substantial epidemiological, clinical, laboratory, and in vitro evidence suggest that dysbiosis can cause loCRC [172,173,174,175,176,177] through epigenetic changes [178]. The most common associations between loCRC and specific bacteria involve *Bacteroides fragilis, Escherichia coli*, and *Fusobacterium nucleatum* [179,180,181,182,183,184,185,186]. However, many factors influence the microbiota composition, including early infant-feeding, diet, and obesity [101,102,187,188]. Even physiological processes can alter the microbiota. In fact, a recent meta-analysis determined that the microbiota composition changes with age, even for healthy individuals [189].

No single study could determine a causative relationship between eoCRC and the microbiota. In a recent small study, the intratumoral microbiome analysis reported a 28% prevalence of *Fusobacterium nucleatum* in samples from eoCRC [190], which was similar to results from loCRC tissues. A similarly small study could not find a significant difference in both the tumor microbial abundance and its alpha diversity between eoCRC and loCRC [191]. However, the potential association between CRC and intestinal dysbiosis might go beyond the link with single pathogens. Diet-mediated alterations could modify CRC risk thanks to metabolic byproducts. Specifically, the consumption of a diet rich in processed meats and poor in vegetables and legumes could promote the growth of sulfur-metabolizing microbes. Their metabolic output is hydrogen sulfide, a carcinogenic compound [192]. This diet, named the “sulfur microbial diet”, was recently associated with increased risk for loCRC in a cohort of older men [193]. Very recently, the Nurses’ Health Study II prospectively confirmed that long-term adherence to such a diet increases the risk of early-onset adenoma. This risk was even higher for lesions with advanced histopathology and those of the ascending [194].

### 7.3. Other Non-Modifiable Risk Factors and eoCRC

Family history remains a substantial risk factor for eoCRC, conferring a relative risk for eoCRC of 4.21 (CI_95%_: 2.61–6.79) [83], and it accounts for up to 20–30% of eoCRC [15,155,156,157,158]. This implies that a substantial fraction of eoCRC is preventable [157], but patients are often non-compliant with the recommendation to start colonoscopy earlier, especially if young [195,196,197]. On the other hand, 16–20% of eoCRC can be attributed to hereditary CRC syndromes [15,156,198,199]. When a pathogenic variant is present, most patients have Lynch syndromes, and the rest may have pathogenic variants in *APC*, *BRCA1*/2, *MutYH,* or other cancer predisposition genes [15,156]. However, genetic studies provide an unsatisfactory explanation for the rising incidence of eoCRC. In fact, under the assumption of Hardy–Weinberg equilibrium, the distribution of genetic traits should not fluctuate with time, unless an evolutionary pressure is present [200].

The interplay between genetic and environmental factors remains elusive. Most hereditary CRC syndromes display a consistent variability in terms of penetrance and expressivity [121,122,123,124,125,126,127]. Some have suggested that diet could explain, at least in part, this degree of variability [201,202,203]. Few exploratory studies have investigated this hypothesis [204,205,206]. Unfortunately, the results have been largely underwhelming. In fact, in patients with Lynch syndrome, the diet seems not to have an association with the age of onset of CRC [205,206]. Perhaps Lynch syndrome confers such a high risk of CRC that the diet is not powerful enough to alter the disease course. Nevertheless, appropriately designed and sufficiently powered studies are still desirable. The risk of CRC is not equally distributed among the four Lynch syndrome genes [207,208,209]. Thus, diet might explain some disease variability in those genes that confer a relatively lower risk of CRC.

## 8. Discussion

Since eoCRCs have a poor family history of CRC and hereditary gastrointestinal tumor syndromes account only for a minority of eoCRC cases, exogenous risk factors need to be investigated to better understand the global eoCRCs’ burden. While consistent data are available on exogenous risk factors involved in loCRC pathogenesis, to date, the corresponding studies on eoCRCs’ populations are still scant, particularly regarding dietary risk factors and physical activity. Indeed, few studies have investigated diet [29,33,37,57,58,59,65] and physical activity [27,28,29,37,57,59,96] as risk factors involved in the pathogenesis of eoCRC and its precursors, with contrasting results. The literature published so far on the role of diet, alcohol, and physical activity involved in eoCRC pathogenesis consists mostly of retrospective and observational studies with evident heterogeneity. Some studies did not involve a population of eoCRCs alone, but included eoCRC precursors, too. While the U.S.A. is the country in which most studies have been conducted, other authors focused exclusively on peculiar dietary and lifestyles while diets from different parts of Europe were not analyzed. Another bias can be identified in the usage of different, not shared questionnaires assessing mostly selected food groups, without analyzing cooking, processing, and storage techniques that might help to explain this increasing eoCRC incidence. Some research was performed on small samples of patients or focused exclusively on a female population, while others were on a male sample. Physical activity was analyzed using different activity or inactivity indexes among all the available studies on eoCRC risk. Finally, only a few studies investigated the presence of hereditary gastrointestinal syndromes, thus not allowing the evaluation of possible interaction with the genetic background.

On the other hand, a growing number of recent, high-quality studies appears to demonstrate a consistent association between high body weight/obesity and eoCRC [26,27,31,70,80,83,84,103,104,105,107,119]. Some limited evidence of no effect [29,30] or even inverse association [32,59,113] also reported in the literature warrants further large and systematic studies to shed further light on the presence of partly conflicting results. Nevertheless, the balance of evidence is heavily in favor of high BMI being a risk factor for the development of eoCRC. Currently available data appear to consistently associate tobacco smoking with an increased risk of eoCRC [26,27,32,59,70,80,84,103], similarly to what is already well established in late-onset disease. A recent prospective multicenter study showed that eoCRCs were more likely to be never smokers compared to loCRCs [113]. This stresses the need for further studies considering current smokers distinctly from former ones, rather than the simple dichotomy never- vs. ever-smoker, and mostly the number of packs/year until now considered only by Chang et al. [59].

The alarming global increase in eoCRCs, highlighted from the international epidemiological studies published so far, requires well-designed international multi-center studies to systematically analyze which exogenous risk factors are responsible for its increase.

An international, multicentre case–control study could be useful to understand the role of diet, alcohol, physical activity, obesity, and smoking habits in eoCRC pathogenesis by comparing eating habits in countries with increasing versus stable or decreasing eoCRC incidence. The consequent identification of an association between eoCRC and specific modifiable risk factors could select areas of future interventions for eoCRC primary prevention. For this purpose, an ad hoc designed, validated semi-quantitative food frequency questionnaire (SQFFQ) associated with a detailed questionnaire on physical activity/inactivity and smoking status needs to be shared among different countries to systematically analyze the exogenous eoCRC risk factors of countries with different eoCRC rates. The SQFFQ has to evaluate a comprehensive list of foods and drinks, focusing on the amount of processed food, types of seasoning, and cooking, too. Indeed, processed foods, whose consumption has drastically increased, ubiquitously contain food emulsifiers known for altering the gut microbiota composition with pro-inflammatory and pro-tumorigenic effects [210,211,212,213,214,215]. Carboxymethylcellulose (CMC) and polysorbate 80 (P80) are some of the most commonly used emulsifiers, a subtype of food additives. Their regular consumption showed to exacerbate colorectal tumor development in a preclinical model of colitis-induced colorectal cancer [215], and to aggravate the initiation and development of genetically driven CRC, as demonstrated in mice mutated for the tumor suppressor gene APC [216]. In this study, the LINE-1 methylation status on formalin-fixed, paraffin-embedded samples of neoplastic colonic mucosa as well as microbiota on stool samples of eoCRC patients could be evaluated in relation to dietary and lifestyle habits, also taking into account the new “age continuum” model proposed by Akimoto et al. for this epigenetic alteration as compared to the sharp distinction between eoCRC and loCRC with the age cut-off set at 50 years [138].

As discussed above, each of these dietary and lifestyle factors leads to an increased risk of CRC through different mechanisms. However, the literature suggested the gut microbiota as the common denominator by which these exogenous risk factors could determine the onset of CRC. To date, several bacteria are involved in CRC tumorigenesis: *Fusobacterium nucleatum* and *Bacteroides fragilis* enriched in CRCs, butyrate-producing bacteria depleted in CRCs, and finally, strains of *Escherichia coli* producing colibactin [175,217,218]. Colibactin is a genotoxin that generates DNA crosslinks and double-strand breaks, causing genomic instability and thus promoting tumorigenesis in preclinical models [219]. Interestingly, colonization of colibactin-producing *E. coli* was demonstrated in 15% of stool samples of three-day neonates, suggesting a potential role in eoCRC pathogenesis [220]. As a distinct microbial signature has recently been identified in Lynch syndrome [221,222], future large studies should analyze the role of microbiota in eoCRC pathogenesis, focusing also on the role of colibactin-producing *E. coli* associated with different dietary habits.

## 9. Conclusions

This systematic review confirms that alcohol and obesity have the strongest association with eoCRC. Additional risk factors for eoCRC include low physical activity, cigarette smoking, sugary drinks, processed meat, and a diet poor in fruits and vegetables.

Epigenetic changes and intestinal dysbiosis are putative mechanisms involved in this global increase in eoCRC, mediating the combination of an unmodifiable predisposition with the exposure to exogenous modifiable risk factors. The association between eoCRC and tumor LINE-1 hypomethylation suggests that exogenous risk factors could fuel eoCRC development through epigenetic changes. Epidemiological studies imply that the first decades of life should receive further investigation. The discovery of new risk factors could be explored in early life, from the gestational age to adolescence, including, but not limited to, early introduction of formula feeding, excessive antibiotics use during childhood or pregnancy, or even gestational diseases (pre-eclampsia or diabetes). The hypothesis that early-life exposure to antibiotics and the resulting dysbiosis could promote eoCRC seems realistic, but so far, it is only speculative. Therefore, sufficiently powered studies should assess not only the impact of intestinal dysbiosis but also the influence of additional risk factors on the microbiota during early life.

Several scientific societies have been calling attention to the increasing incidence of eoCRC for over a decade. It is time for concrete and discrete actions including awareness campaigns. Young adults with alarm symptoms must seek medical advice earlier and general practitioners need to consider CRC in young adults even without a family history of CRC. Identifying the modifiable risk factors of eoCRC will inform educational campaigns on the healthy lifestyle measures (diet, drinking, smoking, and physical activity) to revert the epidemiology of eoCRC.

## Data Availability

The data that support the findings of this study are available from the corresponding author [GMC], upon reasonable request.

## Supplementary Materials

The following are available online at https://www.mdpi.com/article/10.3390/cancers13235933/s1: Table S1: Summary of the revised literature on exogenous risk factors in early-onset colorectal cancer. OR—odds ratio; RR—relative risk; eoCRC—early-onset colorectal cancer; BMI—body mass index; NS—statistically not significant; Uv—univariate analysis; Mv—multivariate analysis.
