# Supplementary material for: The Role of Diet and Lifestyle in Early-Onset Colorectal Cancer: A Systematic Review
Source: Cancers (Basel). 2021 Nov 25;13(23):5933. doi: 10.3390/cancers13235933 (PMC8657307; doi:10.3390/cancers13235933)
Supplement: Supplementary file 1 [file cancers-13-05933-s001.zip › cancers-1458254-supplementary.pdf]

**Table S1.** Summary of the revised literature on exogenous risk factors in early-onset colorectal cancer. OR—odds ratio; RR—relative risk; eoCRC—early-onset colorectal cancer; BMI—body mass index; NS—statistically not significant; Uv—univariate analysis; Mv—multivariate analysis.

| Authors, ref. | Type of study, Country | Population (N) | eoCRC Cases (N) | Controls (N) | Age range/Mean Age ± SD | Sex | Exogenous risk factors analyzed | ORs/HRs/RRs (± 95% CI) for eoCRC (unless otherwise specified) | Notes |
|---|---|---|---|---|---|---|---|---|---|
| **Imperiale *et al.* 2008 [37]** | Retrospective case–control study (multicenter), USA | 74 | 20 ACRN (11 eoCRCs) | 54 age-matched | 28-49 | M/F | ●Dietary factors (1998 Block food frequency survey [84]) ●Alcohol (not further specified) ●Smoking (not further specified) ●Regular exercise (not further specified) ●Weight at 21y ●Waist circumference at 21 y ●Lightest weight since 21y ●Smallest waist circumference since 21y | OR *for ACRN* [Regular exercise] = 0.39 (0.12–1.34); NS Pvalue * [lightest weight since 21y] = **0.049** No differences between case and controls in: (i) any of the 28 preselected dietary factors of the Block food frequency survey; (ii) alcohol use; (iii) cigarette smoking; (iv) weight at 21y (lbs.), | Advanced colorectal neoplasia (ACRN) defined as: adenocarcinoma or polyp with high-grade dysplasia of villous histology or tubular adenoma ≥1cm. * Adjusted for differences in gender. |

| | | | | | | | | | |
|---|---|---|---|---|---|---|---|---|---|
| | | | | | | | | waist circumference at 21 y (in.), smallest waist circumference since 21y. | |
| **Rosato *et al.* 2013 [29]** | Case-control study (multicenter), Italy, Switzerland | 1690 | 329 | 1361 age-matched | 19-45 | M/F | ●Alcohol intake (drinks/week) ●Diet (tertile of intake) ●BMI at 30y (kg/m2), ●Occupational physical activity at 30-39y (mainly sitting, mainly standing, intermediate, heavy, strenuous) ●Leisure time physical activity at 30-39y (h/week) | MvA OR [Alcohol intake ≥14 drinks/w] = **1.56** (1.12–2.16); Ptrend = **0.009** MvA OR [BMI ≥ 25] = 0.91 (0.48–1.73); NS MvA OR [physical activity STRENUOUS] = 1.28 (0.56–2.89); NS MvA OR [Leisure time activity >7h/w] = 0.86 (0.45–1.63); NS MvB OR [Processed meat HIGH tertile] = **1.56** (1.11–2.20); Ptrend = **0.008** | MvA: age, sex, center, study, year of interview, education, family history of CRC, alcohol intake. MvB: as MvA + energy intake. |

| | | | | | | | | | |
|---|---|---|---|---|---|---|---|---|---|
| | | | | | | | | MvB OR [Vegetables HIGH tertile] = **0.4** (0.28–0.56); Ptrend **<0.001**<br>MvB OR [Citrus fruit HIGH tertile] = **0.61** (0.45–0.84); Ptrend = **0.002**<br>MvB OR [Red meat HIGH tertile] = 1.07 (0.79–1.47); NS<br>Mv B OR [Bread and cereals HIGH tertile] = 1.12 (0.76–1.64); NS<br>Mv B OR [Fruit HIGH tertile] = 0.75 (0.54–1.02); NS | |
| **Khan *et al.* 2015 [58]** | Case-control study (single-institution), Pakistan | 222 | 74 | 148 age and sex-matched | 41.47 ± 15.54 | M/F | ●Dietary factors (no/yes)<br>●Smoking (no/yes) | Uv OR [Rice and powder] = **0.24** (0.14-0.44) **<br>Mv OR [Rice and powder] | Uv: univariable unadjusted OR.<br>** significant at 1%. |

| | | |
|---|---|---|
| ●Alcohol (no/yes) | = 0.31 (0.10-1.00); NS<br>Uv OR [Vegetarian] = **0.11** (0.06-0.20) **<br>Mv OR [Vegetarian] = 0.06 (0.02-0.22); NS<br>Uv OR [Refined foods] = **0.01** (0.00-0.04) **<br>Uv OR [High fat diet] = **1.98** (1.13-3.49) *<br>Mv OR [High fat diet] = 1.64 (0.58-4.62); NS<br>Uv OR [Smoking] = **2.12** (1.08-4.17) *<br>Mv OR [Smoking] = 1.27 (0.24-6.85); NS<br>Uv OR [Alcohol intake] =**3.90** (2.00-7.58) **<br>Mv OR [Alcohol intake] | * significant at 5%.<br>Mv OR: adjusted for all independent variables (not listed). |

| | | | | | | | | | |
|---|---|---|---|---|---|---|---|---|---|
| | | | | | | | | =2.97 (0.35-24.93); NS | |
| **Kim *et al.* 2016 [26]** | Retrospective cohort study (Kangbuk Samsung Health Study) (multicenter), South Korea | 70428 | 564 ACRN (25 eoCRC) | 396 late-onset ACRN (29 late-onset CRC) | 18-49 | M/F | ●Smoking (never -former/current), ●Obesity (BMI ≥ 25 (kg/m²) | Mv OR *for ACRN* [Current smoking] = **1.37** (1.15-1.63); Pvalue **<0.001** Mv OR *for ACRN* [BMI obese] = **1.23** (1.03-1.47); Pvalue = **0.021** | Advanced colorectal neoplasms (ACRN) defined as: adenoma ≥1cm, adenoma with any component of villous histology, high-grade dysplasia, or invasive cancer. Mv: adjusted for age, sex, smoking, alcohol, obesity (BMI ≥ 25 kg/m2), triglyceride ≥150 mg/dl, HDL-C <40 mg/dl for men and <50 mg/dl for women, LDL-C, DM, hypertension, insulin, regular exercise, CRC family history, CEA. |

| | | | | | | | | | |
|---|---|---|---|---|---|---|---|---|---|
| **Nguyen et al. 2018 [28]** | Prospective cohort study (The Nurses' Health Study II) (multicenter), USA | 89278 | 118 | NA | 41-47 | F | ●Sedentary TV viewing time (h/week) | Mv RR [ 7.1-14 h/w] =1.12 (0.72-1.75) Mv RR [ >14 h/w] =1.69 (1.07-2.67) Ptrend = 0.03 Mv RR for eo-rectal cancer [ >14 h/w] =2.44 (1.03-5.78); Ptrend = 0.04 | Mv: adjusted for height, family history of CRC, diabetes, screening lower endoscopy within past 10y, lower endoscopy due to other indications within past 10y, smoking, alcohol intake, regular aspirin, NSAID, race, multivitamin, menopausal status, menopausal hormone use, dietary intake (total calories, red meat, fiber, folate, calcium), physical activities, BMI |
| **Glover *et al.* 2019 [84]** | Retrospective analysis of a commercial data- | 8873080 | 1680 | 8871400 NoCRC | 20-39 | M/F | ● BMI (kg/m$^2$) ●Alcohol (not further specified) | Mv OR [smoking] = **2.675** (2.406–2.974); **P < 0.001** | eoCRC = 20-39y individuals with CRC |

| | | | | | | | | | |
|---|---|---|---|---|---|---|---|---|---|
| | base (Explorys Inc., Cleveland, OH), USA | | | | | | ● Smoking (not further specified) | Mv OR [obesity] = **1.819** (1.618–2.044); **P < 0.001**<br>Mv OR [Alcohol] = 0.908 (0.658–1.254); P = 0.56 | NoCRC = 20-39y individuals without CRC<br><br>Exclusion criteria: Crohn's disease, ulcerative Colitis, and family history of colorectal cancer (including patients with familial colorectal cancer syndromes). |
| **Kim *et al.* 2019 [27]** | Retrospective cross-sectional study (Kangbuk Samsung Health Study) (multicenter), South Korea | 72356 | 622 ACRN (14 eoCRC)<br><br>20-29y group: 1 eoCRC<br>30-39 y group: 13 eoCRC | NA | 20-39 | M/F | ●Smoking (never/current or former) ●Alcohol intake (≥20 g/d) ●Obesity (BMI ≥ 25 (kg/m²) ●Regular exercise (moderate or vigorous for ≥3 times/week) ●Abdominal obesity | Mv OR *for ACRN* [20-29 y] [Current or former Smoker] = 0.88 (0.49–2.06); NS<br>Mv OR *for ACRN* [30-39 y] [Current or former Smoker] = **1.30** (1.05–1.61); P = **0.015**<br>Mv OR *for ACRN* [20-29 y] [Alcohol | Advanced colorectal neoplasms (ACRN) defined as: adenoma ≥1cm, adenoma with any component of villous histology, high-grade dysplasia, or invasive cancer.<br>Mv: adjusted for age, sex, |

| | | |
|---|---|---|
| (waist circumference > 90 cm for men and > 80 for women) | intake ≥ 20g/d] = 1.42 (0.78–3.27); NS<br>Mv OR *for ACRN* [30-39 y] [Alcohol intake ≥ 20g/d] = **1.34** (1.10–1.63); P = **0.004**<br>Mv OR *for ACRN* [20-29 y] [Regular exercise] = 0.66 (0.23–1.87); NS<br>Mv OR *for ACRN* [30-39 y] [Regular exercise] = 0.98 (0.75–1.28); NS<br>Mv OR *for ACRN* [20-29 y] [BMI obese] = **2.46** (1.24–4.87); P = **0.01**<br>Mv OR *for ACRN* [30-39 y] [BMI obese] = **1.33** (1.11–1.61); P = **0.003**<br>Mv OR *for ACRN* [20-29 | smoking, alcohol, obesity (BMI ≥ 25 kg/m2), NSAID use, regular exercise. |

| | | | | | | | | | |
|---|---|---|---|---|---|---|---|---|---|
| | | | | | | | | y] [Abdominal obesity] = **2.26** (1.12–4.54); P = **0.023**<br>Mv OR *for ACRN* [30-39 y] [Abdominal obesity] = **1.28** (1.05–1.57); P = **0.014** | |
| **Liu *et al.* 2019 [31]** | Prospective cohort study (The Nurses' Health Study II) (multicenter), USA | 85256 | 114 | NA | 41-47 | F | ●BMI (kg/m²) at 18y<br>●Current BMI<br>●Weight change since 18y | MvA RR [current BMI 25-29.9] = **1.37** (0.81-2.30)<br>MvA RR [current BMI ≥ 30] = **1.93** (1.15-3.25) Ptrend = **0.01**<br>MvB RR [BMI at 18y ≥ 23] = 1.63 (1.01-2.61); NS<br>MvC RR [weight change GAIN 20-39.9 kg] = **1.65** (0.96-2.81)<br>MvC RR [weight | MvA: adjusted for height, history of diabetes, smoking pack-years, alcohol intake, regular aspirin, NSAID, multivitamin, menopausal status, menopausal hormone use, dietary intake (total calories, red meat, fiber, folate, calcium), physical activities. |

| | | | | | | | | | |
|---|---|---|---|---|---|---|---|---|---|
| | | | | | | | | change GAIN ≥40 kg] = **2.15** (1.01-4.55) Ptrend = **0.007** | MvB: as MvA + family history of CRC, screening lower endoscopy within past 10y, lower endoscopy due to other indications within past 10y, weight change since 18y. MvC: as MvA + family history of CRC, screening lower endoscopy within past 10y, lower endoscopy due to other indications within past 10y,BMI at 18y. |
| **Syed et al. 2019 [70]** | Population-based cohort analysis (Explorys database 2012-2016), USA | 68860 | 5710 | 11800420 age-matched controls | 25-49 | M/F | ●BMI (kg/m2) ●Smoking (yes/no) ●Alcohol intake (yes/no) | Mv OR [BMI ≥ 30] = 2.88 (2.74-3.04) Mv OR [smoking] = 2.46 (2.33-2.59) Ptrend < 0.001 | Mv: male gender, Caucasian and African-American race, abdominal pain, rectal pain, altered |

| | | | | | | | | | |
|---|---|---|---|---|---|---|---|---|---|
| | | | | | | | | Mv OR [alcohol use] = 1.71 (1.62-1.80) | bowel function, rectal bleeding, weight loss, family history of cancer, GI malignancy, polyps, smoking, alcohol use, colitis, obesity, hypertension, hyperlipidaemia, polyps. |
| **Breau & Ellis 2020 [80]** | Meta-analysis of 6 epidemiologic studies | NA | NA | NA | < 50 | M/F | ● Cigarette smoking (current smoker if consuming cigarettes on a regular basis)<br>● Alcohol<br>● Obesity (BMI of 25 kg/m$^2$ or greater) | pooled OR [smoking and advanced yCRAC] = 1.56 (1.23-1.98) pooled OR [alcohol and advanced yCRAC] = 1.46 (95% CI 1.27-1.69) pooled OR [obesity and advanced yCRAC] = 1.26 (1.04-1.54). | yCRAC = young-onset colorectal adenomas and cancer, in adults less than 50 years). |

| | | | | | | | | | |
|---|---|---|---|---|---|---|---|---|---|
| **Chen et al. 2020 [105]** | Nested case-control study (IBM MarketScan Commercial Database 2006–2015), USA | 113 million | 4673 | 40832 frequency-matched controls | 18-50 | M/F | ●Metabolic syndrome co-morbidity | Mv OR [Metabolic syndrome] = 1.25 (1.09 to 1.43); Ptrend < **0.001** | Mv: age, sex, duration of insurance enrolment and region, health insurance plan, residence and the following factors between 91 days and 2 years prior to the index dates: outpatient visits, Charlson Comorbidity Index without diabetes, IBD, family history of GI neoplasm, screening colonoscopy, other colonoscopies, faecal occult blood test and regular Rx NSAIDs use. |
| **Gausman *et al.* 2020 [30]** | Retrospective case-control study (single-institution), | 4193 | 269 | 2802 late-onset CRC<br><br>1122 age- | 18-49 | M/F | ●BMI (kg/m$^2$)<br>●Smoking (yes/no) | Mv OR *eoCRC vs age-matched controls* [BMI overweight] | Exclusion criteria: personal history of hereditary CRC syn- |

| | | | | | | | | | |
|---|---|---|---|---|---|---|---|---|---|
| | USA | | | matched controls | | | | = 0.98 (0.95-1.00); NS Mv OR *eoCRC vs loCRC* [BMI overweight] = **0.98** (0.95-0.99); P = **0.04** | drome (documentation of Lynch syndrome, familial adenomatous polyposis or MUTYH-associated poliposis, or pathogenic germline mutations in the mismatch repair or EpCAM genes. BMI overweight: 25-29.9 kg/m² |
| **Holowatyj *et al.* 2020 [96]** | Population-based cohort Study (Centers for Disease Control and Prevention and National Institutes of Health/National Cancer Institute's Surveillance, Epidemiology, and End Results Program), | 28790 | 28790 | NA | 15-49 | F | ●BMI (kg/m²) ●Physical inactivity (no leisure-time physical activity) ●Smoking (daily or ≥ 100 cigarettes over lifetime) | Correlation (p) with geographical hotspot of mortality [physical inactivity] = **0.21;** Ptrend **< 0.01** Correlation (p) with geographical hotspot of mortality [current BMI ≥ 30] = **-0.07;** Ptrend **< 0.01** Correlation | Mv: diagnosis age, race/ethnicity, country of CRC diagnosis, marital status, American Joint Committee on Cancer clinical stage, tumor site, histopathologic subtype, cancer sequence, sur- |

| | | | | | | | | | |
|---|---|---|---|---|---|---|---|---|---|
| | USA | | | | | | | (p) with geographical hotspot of mortality [smoking] = **0.09;** Ptrend **< 0.01** | gery, chemotherapy, and radiation therapy. |
| **Hussan *et al.* 2020 [104]** | Cohort study (Surveillance, Epidemiology, and End Results database), USA | 1272243 | 10178 (Obese) | NA | 20-49 | M/F | ●BMI (kg/m$^2$) at CRC resection | Average annual percentage change (2002-2013) eoCRC diagnosis [BMI ≥ 30] = **+15.3%;** Ptrend = 0.047 | Patient-level variables: age, gender, median household income quartile by zip code, health insurance status. |
| **Krigel *et al.* 2020 [118]** | Retrospective study (monocenter), USA | 4333 | 363 any CRN, of which 48 ACRN (of which 26 eoCRCs) | 3970 | 18-49 | M/F | ● Smoking status (no, yes, unknown) | Mv OR [smoking yes] = 2.17 (0.97-4.85; P = 0.059<br>Mv OR [smoking unknown] = **3.11** (1.57-6.14); P = **0.001** | ACRN = advanced colorectal neoplasia: advanced adenomas or adenocarcinoma.<br><br>Exclusion criteria: personal history of CRN, inflammatory bowel diseases, those undergoing colonscopy for screening |

| | | | | | | | | | |
|---|---|---|---|---|---|---|---|---|---|
| | | | | | | | | | only or without a known indication. |
| **Low *et al.* 2020 [32]** | Retrospective case-control study (Veterans Health Administration), (multicenter), USA | 68067 | 651 | 67416 Age-matched normal Colonoscopy Controls | 18-49 | M/F | ●Smoking (never/former/ current) ●BMI (underweight /normal/overweight/obese/ unknow) ●Aspirin use (no/yes) | Mv OR [Former smoker] = **0.82** (0.60-1.12) * Mv OR [Current smoker] = **1.10** (0.89-1.35) * Mv OR [BMI underweight] = **1.87** (0.86-4.04) * Mv OR [BMI overweight] = **0.69** (0.55-0.87) * Mv OR [BMI obese] = **0.69** (0.55-0.86) * Mv OR [aspirine use] = **0.66** (0.52-0.84) * | * P < 0.05. |
| **Sanford *et al.* 2020 [103]** | Cross-sectional household survey (National Health Interview Survey – self reported diagnosis), USA | 321975 | 239 | NA | 18-49 | M/F | ●BMI (kg/m²) ●Smoking (non smoking / current or former smoker) | Mv OR [BMI ≥ 30, self-reported EOCRC] = **1.39** (1.00–1.92); P = 0.049; Mv OR [Age (< vs. ≥50 years) *BMI | Mv: BMI, age, sex, race, ethnicity, insurance status and smoking. |

| | | | | | | | | | |
|---|---|---|---|---|---|---|---|---|---|
| | | | | | | | | (< vs. ≥30)] = **0.67** (0.47-0.94); P = 0.02 Mv OR [smoking, self-reported EOCRC] = **1.51** (1.10–2.08); P = 0.01 | |
| **Zheng *et al.* 2020 [33]** | Prospective cohort study (The Nurses' Health Study II) (multicenter), USA | 29474 | 375 early-onset high-risk adenoma (eoHRA) | NA | 41-47 | F | ●Western dietary pattern (quartile or quintile of consumption) ●Prudent dietary pattern (quintile of consumption) | Mv OR *for eoHRA* [Western diet HIGHEST quintile] = **1.67** (1.18-2.37); Ptrend = **0.01** Mv OR *for eoHRA* [Prudent diet HIGHEST quintile] = **0.69** (0.48-0.98); Ptrend = **0.03** Mv OR *for advanced adenomas of distal colon and rectum* [Western diet HIGHEST quartile] = **1.65** (1.14-2.38); Ptrend = **0.01** | High-risk adenomas (HRA) defined as any of the following: ≥1 cm in size, tubulovillous/villous histology, high-grade displasia, presence of ≥3 adenomas. Advanced adenomas defined considering only size and histology. Mv: adjusted for age, total caloric intake, time period of endoscopy, n. |

| | | | | | | | | | |
|---|---|---|---|---|---|---|---|---|---|
| | | | | | | | | Mv OR *for advanced adenomas of distal colon and rectum* [Prudent diet HIGHEST quartile] = **0.68** (0.47-0.99); Ptrend = **0.04** | of reported endoscopies, time in years since the most recent endoscopy, reason for the current endoscopy, height, BMI, family history of CRC, menopausal status, menopausal hormone use, type 2 diabetes, smoking pack-years, physical activity, multivitamin, regular use of aspirin and NSAID |
| **Agazzi *et al.* 2021 [117]** | Retrospective observational study (single-center), Italy | 1778 | 223 (196 adenomas, 27 eoCRCs) | 1555 Age-matched Colonoscopy Controls | 18-49 | M/F | ● Smoking (no/previous/current) | Mv OR [current smoking] = 1.10 (0.65-1.86); P = 0.724 Mv OR [previous smoking] = 0.89 (0.50-1.58);P = 0.693 | Exclusion criteria: patients with Lynch syndrome or other familial polyposis syndromes. Multivariable logistic regression was per- |

| | | | | | | | | | |
|---|---|---|---|---|---|---|---|---|---|
| | | | | | | | | | formed pooling together cases of adenoma and eoCRC, considering age as a continuous variable |
| **Archambault *et al.* 2021 [57]** | Data from 13 population-based studies (Colon Cancer Family Registry, the Colorectal Transdisciplinary study, and the Genetics and Epidemiology of Colorectal Cancer Consortium) | 7816 | 3767 | 4049 Age-matched | < 50 | M/F | ● Smoking (pack-years of smoking among current and former smokers) ● Sedentary lifestyle (yes if moderate/vigorous physical activity, leisure time, and undifferentiated activities for < 1 hour/week) ● Alcohol (<1 g/day - nondrinker, 1-28 g/day, > 28 g/day). ● Dietary factors: fruit, vegetable, red meat, processed meat | Mv OR [Pack-years of smoking] = 0.96 (0.92-1.01); P = 0.12 Mv OR [Sedentary lifestyle] = 1.13 (0.88-1.44); P = 0.34 Mv OR [Alcohol use, 0 g/d] = **1.23** (1.08-1.39); **P = 0.001** Mv OR [Alcohol use, >28 g/d] = **1.25** (1.04-1.50); **P = 0.02** Mv OR [Lower fruit intake, servings/dd] = 1.01 (0.96-1.07); P = 0.69 | All self-reported variables were ascertained at the reference time for each study, defined as patient selection or blood collection for cohort studies and 1-2 years prior to selection for case-control studies, to ensure exposures were assessed before cancer diagnoses. For studies assessing height and BMI via direct measurement, variables |

| | | | | | | | | | |
|---|---|---|---|---|---|---|---|---|---|
| | | | | | | | (servings/day); total dietary fiber intake (g/day). | Mv OR [Lower vegetable intake, servings/dd] = 1.00 (0.94-1.06); P = 0.98<br>Mv OR [Greater red meat intake, servings/dd] = **1.10** (1.04-1.16); **P <.001**<br>Mv OR [Greater processed meat intake, servings/dd] = 1.03 (0.95-1.12); P = 0.43<br>Mv OR [Lower total fiber intake, g/dd] = 1.11 (1.00-1.23); P = 0.06 | were captured at the reference time of each respective study. |
| **Chang *et al.* 2021 [59]** | Retrospective case-control study (Ontario Cancer Registry), Canada | 428 | 175 | 253 age and sex-matched | 20-49 | M/F | ● BMI (kg/m$^2$) 2 years before diagnosis (obese ≥ 30.0 kg/m2)<br>● BMI at 20years | Mv OR [BMI obese, 2y before] = 0.59 (0.34–1.01); *p*trend = 0.06<br>Mv OR [BMI obese, at 20y] = 0.43 | Information on tumor microsatellite instability (MSI) status was obtained from pathology reports of |

| | | | |
|---|---|---|---|
| | ● Smoking status (never, ever)<br>● Pack-year smoking (<3.5 / 3.5-9.9 / ≥10)<br>● Alcohol (<once per month / 1-3 times per month / 1-6 times per week / daily)<br>●Physical activity (active: ≥ 24 units; somewhat active: 14–23 units; insufficiently active: < 14 units)<br>●Sedentary time (<5h per day / 5-10h per day / ≥ 10h per day)<br>●Diet (daily or weekly frequency of consumption of fruits, vegetables, high-fiber /wholegrain | (0.20–0.90); *p*trend = 0.06<br>Mv OR [ever smoking] = 1.07 (0.68–1.67)<br>Mv OR [smoking, first tertile of pack-years] = 1.94 (1.04–3.60)<br>Mv OR [smoking, third tertile of pack-years] = 0.79 (0.40–1.57)<br>Mv OR [sedentary time, ≥ 10 h/day] = **1.93** (1.02–3.65); ***p*trend = 0.049**<br>Mv OR [physical activity, insufficiently active] = 1.46 (0.90–2.38)<br>Mv OR [daily alcohol consumption] = | eoCRCs: 89% MSI-low, 11% MSI-high.<br>Dietary habits and other lifestyle factors were referred to the time period “2 years ago”.<br>Mv: Adjusted for age, sex, family history of CRC, regular aspirin/NSAID use, smoking, physical activity, BMI, alcohol, red/processed meat intake, total fruit and vegetable intake, high-fiber food intake, calcium supplement use.<br>●Ever smoked: ≥ |

| | | | | | | | |
|---|---|---|---|---|---|---|---|
| | | | | | foods, red meat, processed meat, sugary desserts, fast food, canned food, processed snacks)<br>●Westernized dietary pattern (quartile)<br>●Drinks (sugary drinks, coffee/ tea, water, sugar substitutes) | 1.07 (0.49–2.32)<br>Mv OR [sugary drinks, ≥ 7/week] = **2.99** (1.57–5.68); ***p*trend = 0.002**<br>Mv OR [sugary desserts, (3-6 times/week] = 2.28 (1.28–4.04)<br>Mv OR [Total fruits and vegetables, ≥ 6 servings/day] = 0.58 (0.30-1.13);*p*trend= 0.11<br>Mv OR [red meat, ≥ 5 servings/week] = 1.06 (0.56-1.98)<br>Mv OR [processed meat, ≥ 3 servings/week] = 1.23 (0.62-2.42)<br>Mv OR [sugary dessert, ≥ 7 | 100 cigarettes before 2 years ago.<br>●Pack-year: calculated by multiplying the number of packs of cigarettes smoked per day (1 pack = 20 cigarettes) by the number of years smoked<br>●Physical activity: adapted from the Godin-Shephard Leisure-Time Physical Activity Questionnaire: number of times engaged in strenuous or moderate exercise for > 15 min during a typical 7-day week. Weekly frequency (times per |

| | | | | | | | |
|---|---|---|---|---|---|---|---|
| | | | | | | times/week] = 1.45 (0.86–2.47) Mv OR [fast food, ≥ 2 times/week] = 1.84 (0.98–3.46); *p*trend=0.07 Mv OR [canned food, ≥ 3 times/week] = 1.70 (0.95–3.05); *p*trend=0.09 Mv OR [coffee or tea, ≥ 3 cups/day] = 1.68 (0.85–3.30) Mv OR [Artificial sweeteners, ≥ 7 times/week] = 1.66 (0.89–3.13) Mv OR [Western diet, quartile 4] = **1.92** (1.01–3.66); ***p*trend = 0.047** | week) of strenuous and moderate exercise was multiplied by 9 and 5, respectively, and summed across activity types to calculate the composite score (active: ≥ 24 units; somewhat active: 14–23 units; insufficiently active: < 14 units) ●Sedentary time (number of hours spent sitting on a typical day at work, school, home, in a car/bus/train, and during leisure time, averaged over weekdays and weekend |

| | | | | | | | | | |
|---|---|---|---|---|---|---|---|---|---|
| | | | | | | | | NB: p value for linear trend calculated by treating the ordinal variable or the median value of each category (where applicable) as a continuous variable in the model, shown only when ptrend is < 0.10 for at least one of the age- and sex- adjusted and multivariable-adjusted ORs | days) supplement use |
| **Himbert *et al*. 2021 [113]** | Prospective cohort study (ColoCare Study) (multicenter), USA and Germany | 2193 | 459 eoCRCs | 1734 loCRCs | 60 ± 13 | M/F | ●BMI (underweight ≤18.5 kg/m2; normal weight >18.5 to <25 kg/m2; overweight ≥25 kg/m2 to <30 kg/m2; obese ≥30 kg/m2). | Mv OR [BMI overweight] = 0.56 (0.41-0.76) Mv OR [BMI obese] = 0.66 (0.48-0.90) Mv OR [Never smoker] = 1.55 (1.21–1.98) | MV: adjusted for sex, race, tumor site and stage, BMI, smoking, and study site. |

| | | | | | | | | | |
|---|---|---|---|---|---|---|---|---|---|
| | | | | | | | ● Smoking (ever-smoker/never smoker) | | |
| **Hur *et al.* 2021 [65]** | Prospective cohort study (The Nurses' Health Study II) (multicenter), USA | 95464. Only 41272 reported adolescenceintake at age 13–18 years | 109 eoCRCs | NA | 41.5 ± 5.5 | F | ● Sugar-sweetened beverages (SSBs) during adulthood (validated food frequency questionnaires -FFQ- every 4 years ● SSBs intake during adolescence (FFQ in 1998) | Mv RR [≥2 servings of SSBs per day *in adulthood*] = **2.18** (1.10-4.35); Ptrend=**0.02** Mv RR [≥2 servings of SSBs per day *in adolescence*] = **3.41** (1.08-10.8); Ptrend=**0.01** | MV: adjusted for race, height, BMI, menopausal status and menopausal hormone use, family history of CRC, pack-years of smoking, physical activity, regular use of aspirin, regular use of non-steroidal anti-inflammatory drugs, current use of multivitamins, intake of alcohol, red and processed meat, dietary fibre, total folate and total calcium. |

| | | | | | | | | | |
|---|---|---|---|---|---|---|---|---|---|
| **Li *et al.* 2021 [107]** | Meta-analysis of 4 epidemiologic studies | NA | NA | NA | ≤ 55 | M/F | ● BMI ($kg/m^2$) (before diagnosis but not near diagnosis) | pooled OR [BMI ≥ 25 $kg/m^2$ and CRC ≤55y] = 1.42 (1.19-1.68); I(2) = 0%, Pheterogeneity = 0.60 pooled OR [BMI ≥ 25 $kg/m^2$ and CRC <50y] = 1.38 (1.08-1.76) pooled OR [BMI ≥ 30 $kg/m^2$ and CRC ≤55y] = 1.88 (1.40-2.54); I(2) = 92%, Pheterogeneity < 0.01) pooled OR [BMI ≥ 30 $kg/m^2$ and CRC <50y] = 1.87 (1.31-2.68) | Exclusion criteria: studies only examined risk factors for advanced polyps or combined advanced polyps with CRC in the outcome definition. |
| **O'Sullivan *et al.* 2021 [83]** | Meta-analysis of 14 epidemiologic studies | NA | NA | NA | < 50 | M/F | ● BMI ($kg/m^2$) ●Alcohol (high vs. non-drinkers) ●Cigarette Smoking | Pooled RR [obesity] = 1.54 (1.01-2.35), I2 = 98.7% p=0.00 Pooled RR [alcohol] = 1.71 (1.62- | Exclusion criteria: studies only examined risk factors for advanced polyps or combined |

| | | | | | | | | | |
|---|---|---|---|---|---|---|---|---|---|
| | | | | | | | | 1.80); I2 = 0.0% p= 0.85 Pooled RR [smoking] = 1.35 (0.81-2.25), I2 = 99.4% p= 0.00 | advanced polyps with CRC in the outcome definition. |
| **Schumacher *et al.* 2021 [119]** | Retrospective population-based case–control study (Kaiser Permanente Southern California), USA | 6160 | 1032 | 5128 Age and sex-matched | 15-49 | M/F | ● BMI (kg/m$^2$), ≥1 year prior to eoCRC diagnosis /index date ● Smoking (current/former/never) | Mv OR [obesity BMI ≥ 30] = **1.41** (1.15–1.74); **P <0.01** Mv OR [obesity BMI ≥ 30 and colon cancer] = **1.56** (1.17–2.07); **P <0.01** Mv OR [obesity BMI ≥ 30 and rectal cancer] = 1.19 (0.85–1.68); P = 0.31 Mv OR [current smoking] = 0.82 (0.62-1.07) Mv OR [former smoking] = 0.78 (0.63–0.97) | Exclusion criteria: history of inflammatory bowel disease. Mv adjusted for race/ethnicity, smoking, family history, neighborhood socioeconomic status, and health care utilization. |
